# The low impact of fish traps on the seabed makes it an eco-friendly fishing technique

**DOI:** 10.1371/journal.pone.0237819

**Published:** 2020-08-21

**Authors:** Dorothée Kopp, Yann Coupeau, Benoît Vincent, Fabien Morandeau, Sonia Méhault, Julien Simon

**Affiliations:** IFREMER, Unité de Sciences et Technologies Halieutiques, Laboratoire de Technologie et Biologie Halieutique, Lorient, France; Instituto Portugues do Mar e da Atmosfera, PORTUGAL

## Abstract

Besides understanding the effects of fishing on harvested fish stocks, effects on non-target species, habitats and seafloor integrity also need to be considered. Static fishing gears have often been mentioned as a lower impact fishing alternative to towed gears, although studies examining their actual impact on the seafloor are scarce. In this study, we aimed to describe fish trap movements on the seafloor related to soaking time and trap retrieval. Impacts on the seafloor of lightweight rectangular traps and heavier circular traps were compared. We used 3D video cameras to estimate sweeping motion on the seabed and penetration into the sediment during soaking time. The area and distance swept by each type of trap during retrieval was determined by a camera set up facing the sea bottom. The potential rotation of the traps around the mainline was assessed using an Acoustic Doppler Current Profiler. Results showed that no penetration and almost no movements could be detected during soaking time for either lightweight or heavy commercial traps, even for high tidal coefficient (maximum 6 cm). No rotation could be observed when the tide turned. The swept area covered by a trap during retrieval was low (maximum 2.04 m^2^) compared to towed fishing gear and other static gear.

## 1. Introduction

Understanding the impacts of fishing goes beyond looking at its effects on harvested fish stocks [1,2], as it is also necessary to consider the impacts of fishing on non-target species [3–5], as well as on habitats and seafloor integrity [6,7].

Sea floor integrity is one of 11 ecosystem descriptors for which the European Marine Strategy Framework Directive (MSFD; 2008/56/EC) aims to evaluate the Good Ecological Status (GES). For this descriptor, GES is defined as “Sea-floor Integrity is at a level that ensures that the structure and functions of the ecosystems are safeguarded and benthic ecosystems, in particular, are not adversely affected” [8].

Most studies on seafloor integrity in relation to fishing activities concern mobile bottom- contacting gear such as bottom trawls, dredges or bottom seines [9]. These gears are known to have biological effects by killing or damaging organisms [10,11], but also by modifying richness, biomass or productivity of the benthic communities [12]. Geochemical effects such as modification of biogeochemical fluxes due to penetration of the gear into the sediment [13] or physical effects (e.g., sediment resuspension or modification of seabed topography) have also been reported [14,15]. A meta-analysis performed in the UK, considering 31 habitats and 14 categories of fishing activities and based on sensitivity and resilience scores, revealed that most habitats were highly sensitive to towed fishing gears such as beam trawls and scallop dredges, whereas only three habitats were sensitive to potting and only at heavy fishing intensity [16]. The same observation was made in the north-east USA [17], where habitat vulnerability was high for dredges and otter trawls whereas sensitivity was much lower for fixed gears such as gillnets, traps and longlines.

Static fishing gears are often mentioned as lower impact alternatives to towed gears, although studies conducted on their impact on the seafloor are scarce (but see [18]). Thus, among 130 studies on static gears [19], impacts were reported on discard (85 papers) and escapee (8) mortality, depredation (10) and ghost fishing (24), but only three studies concerned mortality due to habitat degradation by fishing activities [20–22]. The few studies on set gillnets, traps or pots mainly concerned changes in species composition of the benthic community, and results differ according to the habitat considered. For example, lobster traps set on coral reef flats could reduce the percentage of benthic cover from 45% to 31% in quadrats along the trap movement path [22], whereas in temperate rocky habitats [23] no assemblage changes were observed between areas subject to potting compared with non-fished areas. In subtropical kelp forests and rocky reefs, set gillnets damaged or removed ~19% of all kelp and ~17% of all gorgonians within 1 m of the net path whereas traps had almost no impact on benthic communities [24].

The sweeping movements of static gear and modifications that this could cause to the substrate (e.g., sediment resuspension or modification of seabed topography) are rarely considered, although a recent study on bottom set gillnets revealed that sweeping movements could be up to 2 m [25]. One study reported trap movement according to the wind during the storm season in the Caribbean [22] and showed that single, buoyed traps could move a mean distance of 3.6 m, affecting an area of 4.6 m^2^ when the trap was set in low depths.

To the best of our knowledge, no studies have yet been made on the physical impact of fish traps rigged in lines even though, in commercial fishing conditions, this gear configuration can consist of up to several hundred metres of leaded rope and be composed of several hundred traps (up to 800 lobster traps in the Gulf of Maine) [26]. In fishing areas subject to tidal currents or high swell, the rope is usually attached to anchors at its extremities to prevent drifting. According to several authors, the traps, the rope or the anchor could all induce injuries at different times during the fishing process e.g., initial setting, changing tides, sliding during retrieval [22,27].

In this study, we aimed to compare a lightweight trap and a heavy trap according to four indicators describing seafloor impact. We used an Acoustic Doppler Current Profiler to estimate trap rotation during tidal current changes, and 3D video cameras to estimate potential sweeping motion on the seabed and penetration into the sediment during soaking time. Video was used to estimate trap sliding during retrieval. Each indicator was recorded under strong and low tidal currents. The results obtained may be considered as a case study of trap physical impacts on the seabed.

## 2. Material and methods

### 2.1. Sea trials

The sea trials were performed in the Bay of Quiberon (47°32 N, 003°05 O, Bay of Biscay, East Atlantic, ICES Subarea VIIIa) in July 2019. Two types of commercially-available Norwegian traps, distributed by Fiskevegn, were tested because they differ in weight: a lightweight trap of 13 kg and a heavy one of ~31 kg (referred to hereafter as the lightweight and heavy configurations). The lightweight trap has a rectangular base of 1.50 m * 1 m and is composed of two chambers of 60 cm height. The heavy trap has a truncated conical shape with a round base of 1.40 m diameter and 0.85 cm diameter at the top. It is composed of a single chamber 1.1 m height (S1 Fig). The light traps are used in the 8a ICES area and the main species caught in commercial conditions are conger, ling, edible crab and bib with respectively 758 tons, 4 tons, 3.2 tons and 2 tons in 2019 (SACROIS algorithm, [28]). The mean soaking time in this area is comprised between 12 and 48 hours according to field survey with fishermen. The heavy traps are mainly used in Norway and target cod and tusk.

**Fig 1:**
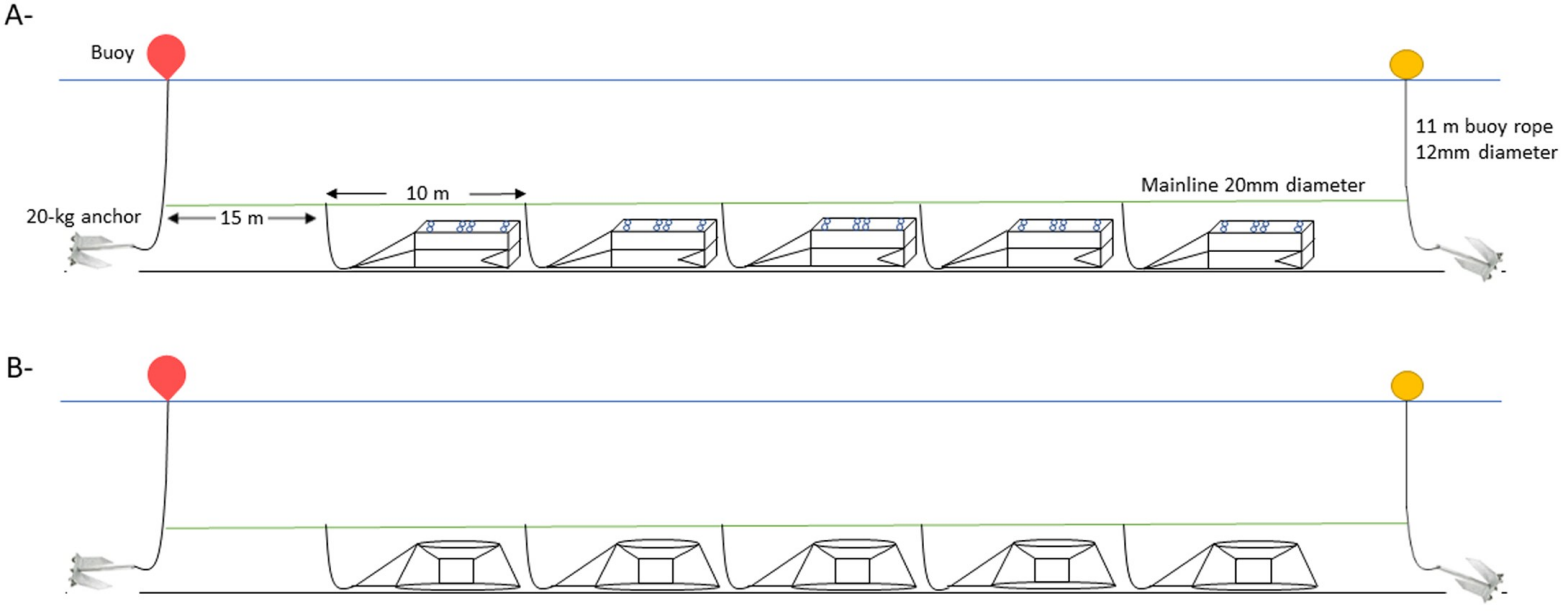
The two configurations tested. A- fishing line of lightweight traps, B- fishing line of heavy traps.

A fishing line with 5 traps was deployed, with approximately 10 m of 20 mm diameter foot rope between each trap (total weight of the rope: 14 kg). The traps were attached to the mainline by a 2 m branchline attached to the bottom frame opposite the trap entrance. At both ends of the mainline there was a 20 kg anchor and a rope attached to a surface buoy (Fig 1). The total weights were 119 kg for the lightweight configuration and 209 kg for the heavy one.

The trap line was soaked in waters no deeper than 12 m in an area of muddy sand seabed for an 8-hour period including a change of tide. Each configuration was soaked twice for both high (between 72 and 104) and low (between 39 and 54) tidal coefficients (hereafter Days 1 & 2 refer to lightweight trap, low tidal coefficient; Days 3 & 4: lightweight trap, high tidal coefficient; Days 5 & 6: heavy trap, low tidal coefficient; Days 7 & 8: heavy trap, high tidal coefficient). The trap line was set in the same direction as the predominant current direction i.e., on a North/South axis (determined by a pre-study in the sampling area–S2 Fig).

### 2.2 Video data collection

#### 2.2.1 Stereovision during soaking time

**Fig 2:**
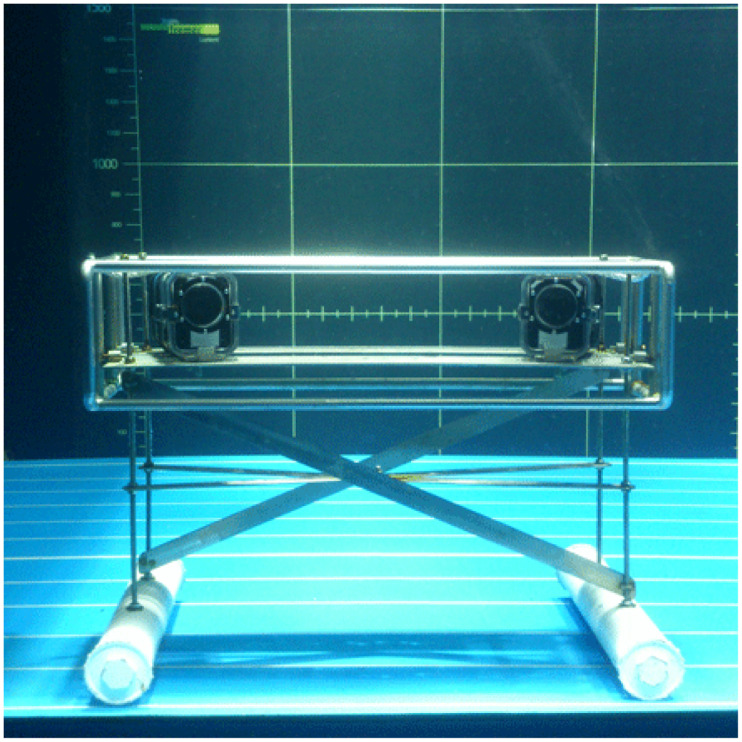
The observation unit in the IFREMER Lorient flume tank.

The experimental setup for stereovision was based on the methodology developed by Savina et al. [25], with an observation device composed of two GoPro Hero 7 cameras mounted on a metal frame weighted with two ballasts of ~8 kg each. The cameras were positioned 60 cm from each other and kept in waterproof housings (Fig 2).

**Fig 3:**
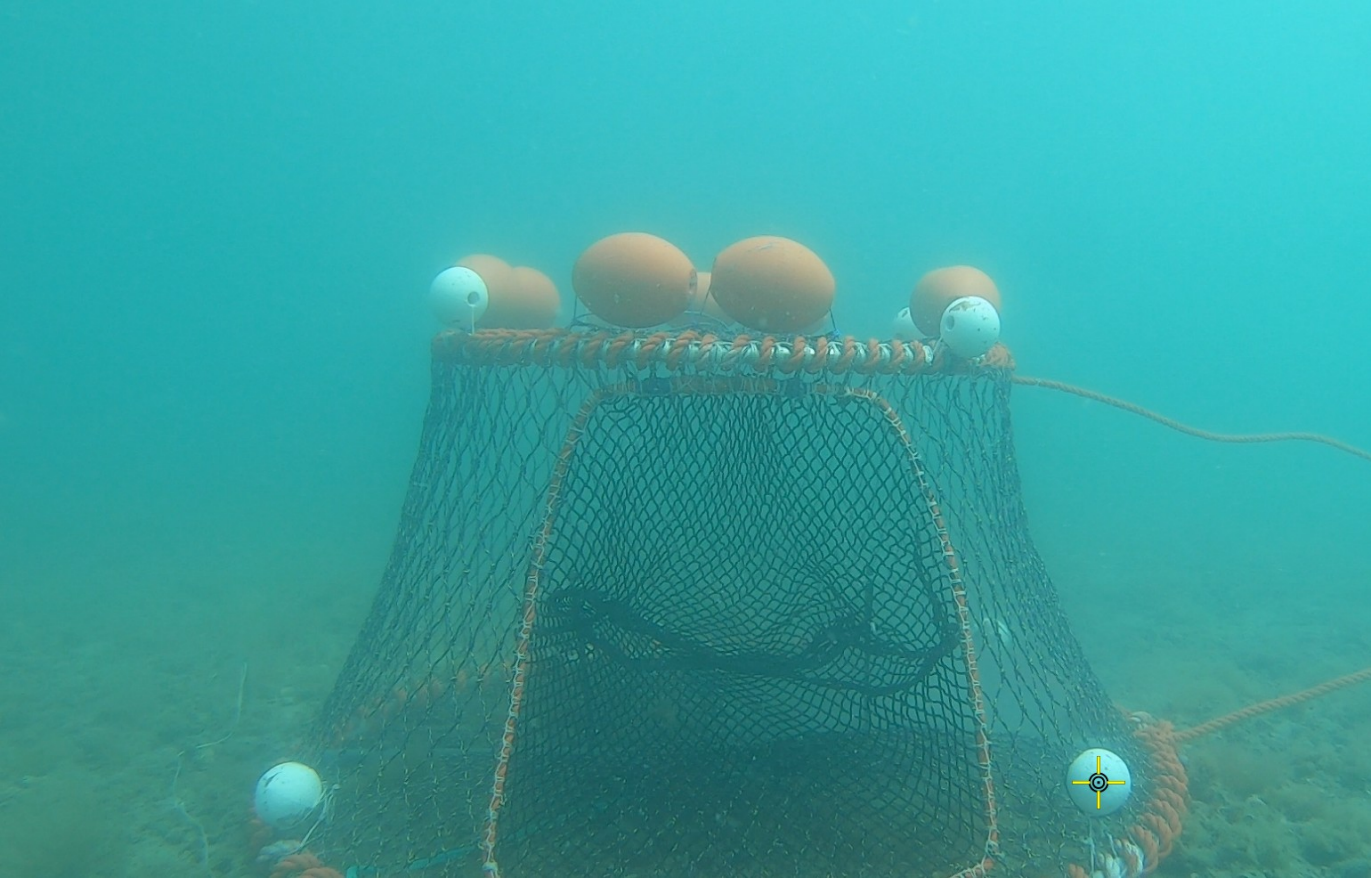
A heavy trap with white buoys attached to create reference points for video analysis.

Before conducting experiments at sea, we tested the grounding of the observation device in high currents in the flume tank at Ifremer Lorient [29]. At sea, the stereovision device was positioned in front of the fourth trap along the line, at about 2 m from the trap. White buoys were added to the trap as reference points for the subsequent video analysis (Fig 3).

Settings were identical for the two cameras. The video resolution was set at 1920*1080 pixels and frames per second was set at 24. Left and right cameras were synchronized using a light flash. The videos were analysed using the free open-source program VidSync (www.vidsync.org). The mathematics of the 3D measurements by this software are fully described in [30]. The different steps of recording were: i) creation of a project in VidSync, ii) importation of the right- and left-hand videos, iii) synchronization of the videos, iv) importation of the files related to the distortion and 3D calibrations, and v) measurement of an identified point on each video (a white buoy in our case) every 10 minutes for a maximum of 8 hours of video recording. Before accurate 3D measurement of trap position could be done, calibration and lens distortion were obtained using a calibration cube and chessboard (S3 Fig). To convert measurements from the videos (units in pixels) to real world measurements (units in metres), a calibration was performed using a 3D calibration frame (S3b Fig) on an in-situ video with sufficient underwater visibility. The 3D calibration frame was composed of white points of known positions. These white points were then tagged in VidSync. To correct the lens distortion, a chessboard (S3a Fig) was positioned in front of the two cameras.

The calibration and distortion corrections were validated for each specific configuration, i.e., camera settings and relative position and orientation. Thus, to ensure that the calibration and distortion corrections were always valid between two sets of measurements, 3D printed parts were used to put the cameras in exactly the same relative position and orientation. In addition, before each experiment (except for Days 3 and 4), three verification measurements were made with points 300 mm apart within each axis, using the calibration frame. From these measurements, the mean and the standard deviation were calculated for each day and each axis (S1 Table). Overall relative errors were smaller than 10%, i.e. 3 cm corresponding to the precision of our experimental setup.

#### 2.2.2 During retrieval

**Fig 4:**
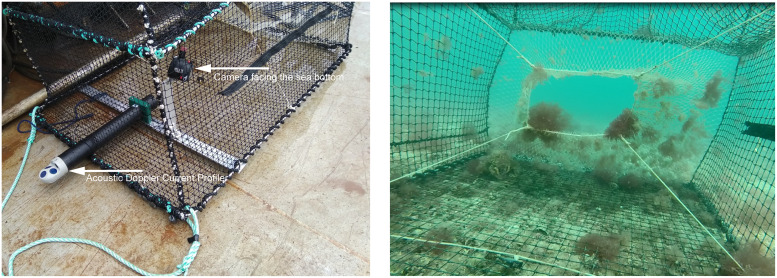
Position of the camera facing the sea bottom and of the Acoustic Doppler Current Profiler (left panel) and View of the bottom from the camera set to quantify the swept distance during retrieval (right panel).

To quantify the swept distance and area of a trap during retrieval, a camera was set up facing the sea bottom (Fig 4). The distance was determined by counting the number of meshes (knowing mesh size) until the trap came away from the bottom using references points on the sea bottom (stones, shells). The corresponding area was calculated by multiplying this distance by the maximum width of the trap.

### 2.3. Current measurements and trap orientation

A Nortek Aquadopp ADCP (Acoustic Doppler Current Profiler) was attached in the lower part of the fifth trap along the line to measure the local absolute water current intensity and direction (Fig 4). The ADCP was set to measure current in 20 cells of 0.15 m to get the current profile from the seabed up to 3 m in height. The first 1.50 m starting from the seabed were not considered, so as to avoid measuring the current beyond any possible wake from the trap. Only the average current in the profile from 1.5 m to 3m was considered. Thus, the measured water current was probably greater than the current at the level of the trap due to the velocity gradient near the seabed. The water velocity during the experiment varied from 0.0008 m.sec^-1^ to 0.3 m.sec^-1^ with a mean current higher at high tidal coefficients (~0.1m.sec^-1^) than at low tidal coefficients (~0.0 5 m.sec^-1^). To assess trap rotation rate, the magnetic orientation (0° for North to 360° turning clockwise) of the trap was measured by the ADCP integrated magnetometer.

## 3. Results

### 3.1 Impacts during soaking time

#### 3.1.1 Trap rotation

**Fig 5:**
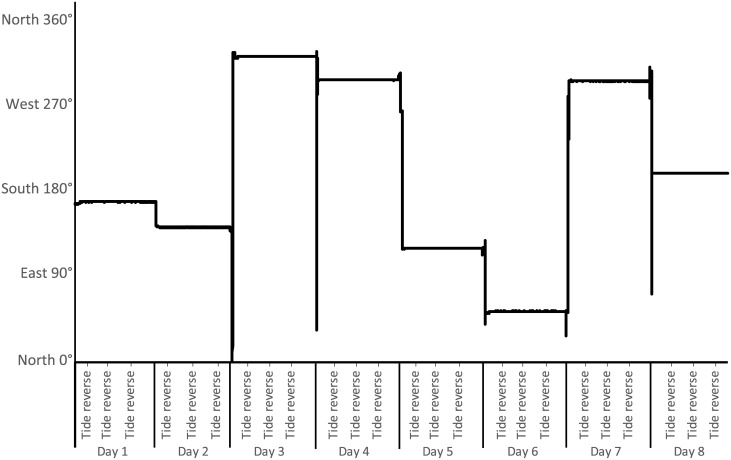
Trap orientation (in degree) during the experiment.

Orientation measures from the ADCP were based on a magnetic compass giving continuous values with 0° for North, 90° for East, etc … Thus, a constant value means that the orientation remains constant. During soaking time, even at the tide change, the orientation of the trap did not vary, meaning that no rotation around the branchline could be detected (Fig 5, S2 Table).

#### 3.1.2 Trap slide and penetration

**Fig 6:**
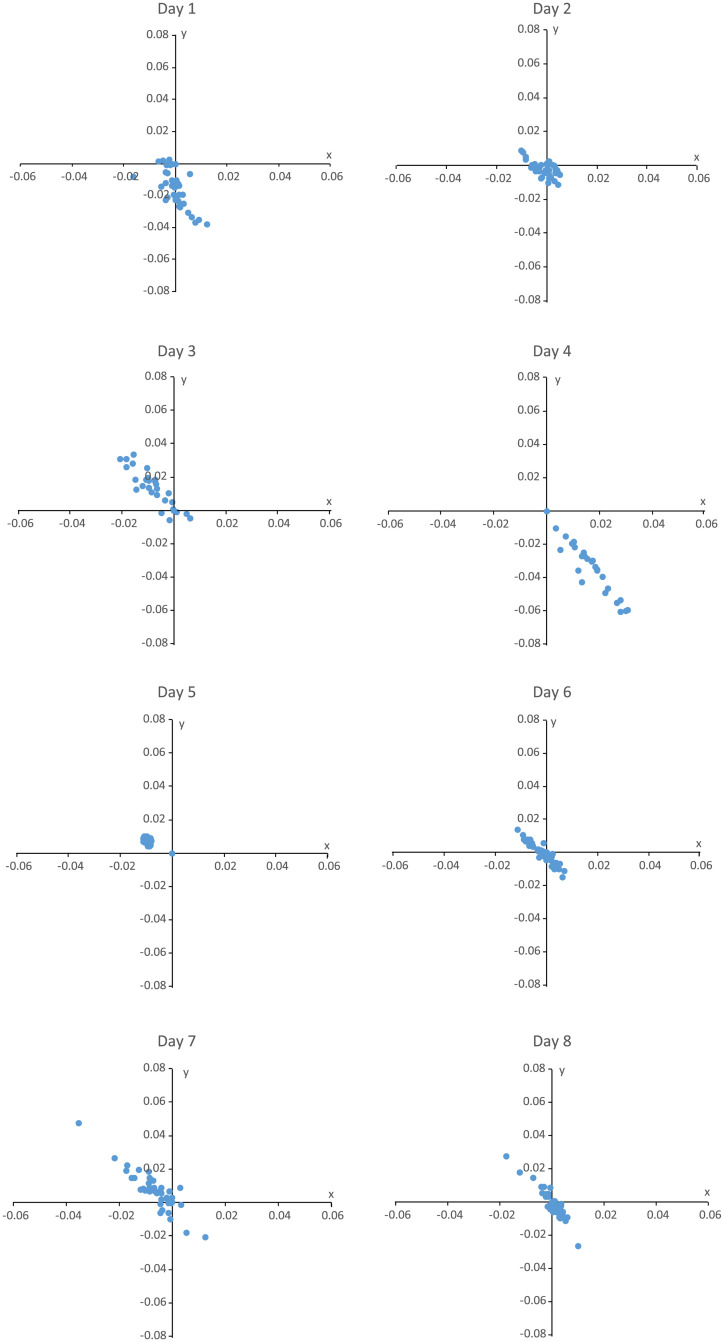
Relative positions of the trap from the initial position (in metres) in the X and Y dimensions.

The stereovision system indicated that penetration into the sediment (z-axis up/down) of either the light or heavy trap ranged between 0.02 cm and 1 cm, which was lower than the potential error of measurement. No larger penetration was observed during the experiment. The maximum distance covered by the lightweight trap on the x-axis (right/left) during low tidal coefficients was 1.6 cm (Fig 6, S2 Table). During high tidal coefficients, the maximum distance covered by the lightweight trap was 3 cm. For the heavy trap, the distance covered during low tidal coefficients could reach 1 cm, and during high tide coefficients 3 cm. On the y-axis (front/back), the maximum distance covered by the lightweight trap during low tidal coefficients was 4 cm. For high tidal coefficients, the maximum distance covered by the lightweight trap was 6 cm. Considering the heavy traps, the distance covered on the y-axis could reach 1 cm during low tidal coefficients, and up to 5 cm during high tidal coefficients.

### 3.2. Impacts during retrieval

For the lightweight configuration, the retrieval phase lasted for a mean of 20 ± 11 seconds. The mean distance swept by the trap was 67 ± 45 cm, with a minimum distance of 25 cm and maximum distance of 136 cm. The corresponding swept areas were 0.375 m^2^ and 2.04 m^2^, respectively. For the heavy configuration, the retrieval phase lasted for a mean of 21 ± 13 seconds. The mean swept distance of a heavy trap was 78 ± 49 cm with a minimum distance of 11 cm and maximum distance of 145 cm. The corresponding swept areas were 0.15 m^2^ and 2.04 m^2^, respectively.

## 4. Discussion

In a review on ways of lowering the impact of fishing activities in the future, fish traps are identified as offering attractive characteristics in terms of both sustainable development and economic viability [26]. Trap fisheries have low fuel needs, the survival of unwanted bycatch species is high and, depending on soaking time and depredation, the traps may capture fish alive and uninjured, meaning that fishermen could obtain a higher selling price. In our case study, we provide information on trap motion during soaking time and retrieval that confirms the low impact of this fishing technique on the seafloor. No movement could be detected during soaking time for both types of trap and so whatever the strength of the current. The swept area was below 2m^2^ (2.10^−6^ km^2^) during retrieval, which is very low compared with other fishing gears. Thus, for different towed fishing gears, the hourly swept area varied from a maximum of 1.6 km^2^ for Scottish seine to ~0.2 km^2^ for beam trawlers [31]. The same order of magnitude was reported for beam trawlers towing two 12-m width beams with a substratum impact of 0.27 km^2^ [13]. The swept area for an encircling trammel net [32], was found to be between 0.180 and 0.654 and km^2^ [33]. Concerning static fishing gear, a swept area of about 0.04 km^2^ for lightweight gillnets and 0.01 km^2^ for heavy gillnets deployed in the Kattegat [25]. It is important to compare these swept areas in light of the catches’ weight. In 2019 in the ICES division 8a where we conducted our study, for one hour bottom trawling, that is one hour of impact on the seafloor, the mean weight of fish catches was 55.1 kg/hour (SACROIS algorithm, fishing gear OTT and OTB, [28]). In the same area for fish trapping (fishing gear FPO), the mean catches’ weight for a fishing sequence of 18 hours (mean soaking time) was 809 kg meaning that the weight per hour fishing is lower. As the traps impact the seafloor solely during gear retrieval that last for few minutes or seconds, it appears that fish trapping is a good compromise between fish catches weight and gear impact. Comparing bottom trawling and creel *Nephrops* fishery in the Kattegat and Skagerrak, [18] also showed that creeling has a reduced seafloor pressure per landed kilo of *Nephrops*. The authors showed that the average swept area per tonne of *Nephrops* landed by creels is comprised between 0.003 and 1.3 km^2^/tonne depending on the extent of dragging and drifting when retrieving the creels. In contrast for trawling, it varies between 21 and 40 km^2^/tonne depending on whether a sorting grid is employed or not.

The severity of fishing gear impact was also examined by [31], who quantified this impact at both the surface and subsurface of the sediment. These authors reported that scallop dredges, flatfish beam trawls and *Nephrops* trawls had the most impact on the subsurface, with a maximum hourly swept area of 0.3 km^2^ for *Nephrops* trawlers. In the present study, no penetration of the trap into the subsurface could be detected, meaning that the impact of trap on subsurface organisms during soaking time is probably inexistent. The movement of the trap during retrieval could however causes damages to sessile organisms at the sediment surface [22] even if in some studies traps have minimal [24] or no [23] impacts on fixed species such as gorgonians, sponges, or algae. This impact on epibenthic organisms was not assessed in the present study.

Even if trap impact on the sea bottom is already quite low, technical solutions can be proposed to further reduce any potential effects. Thus, floating traps are used in cod fisheries in Norway and Sweden [34,35]. Besides reducing the impacts on the seafloor, floating traps prevent the catch of non-target crustaceans and are also more efficient, as the entrance always faces the currents resulting in higher cod catches.

Other issues relating to fish traps could be raised, such as ghost fishing [36] and pollution from traps lost as sea [37,38], but these impacts also exist for other gear types that have higher impacts on the seabed (e.g., gillnets or trammel nets, [39–41]; or trawls, [42]). Some solutions have been proposed to mitigate these impacts: the use of natural fibres [43] or biodegradable plastics in some parts of the trap [44] to disarm the gear if it is lost. This solution allows any captured animals to escape and may provide habitat complexity or refuges for some species, when the natural structures may have been compromised by other fishing gears with a more destructive character [45,46]. Some traps are constructed completely out of in biodegradable plastics that are entirely degraded after a certain time period but that have the same fishing efficiency as commercial traps made of polyethylene [47].

To the best of our knowledge, this study is one of the first attempt to characterize the impacts on the seafloor of two distinct fish traps rigged in lines. The used methodology proves to be an effective tool to evaluate the potential of static fishing gear on the seafloor [25]. These results are specific to the fishing conditions we experienced while taking the measurements in terms of currents, tidal coefficient, sediment type and depth and could thus be subject to variations if the traps were set in different environments. Our experimental setting was composed of 5 traps but it is important to notice that for most of the fleets operating fish traps, several ten of traps could be deployed and even several hundred for crustaceans or cephalopod pots. Nonetheless, our results underline the potential of fish traps as a promising type of fishing device for sustainable development of marine ecosystems. With the emergence of offshore windfarms and tidal energy or the implementation of Marine Protected Areas, stakeholders need to find trade-offs between fishing activities, renewable energy generation and biodiversity conservation and trap fishing has been remarked as a promising fishing [23,48–50]. In the Bay of Biscay, fish traps are mainly used by small scale fisheries. They are usually used together with other gear types along the year, depending on fish seasonality. In that way, fish traps contribute to gear diversity within the coastal zone, which mitigate the pressure on the seafloor.

## Supporting information

S1 Fig Current directions in degree measured prior the trap impact experiment to determine the settlement direction of the trap line. (PDF)

S2 Fig (DOCX)

S3 Fig (DOCX)

S1 Table (XLSX)

S2 Table (XLSX)
