# Supplementary material for: The low impact of fish traps on the seabed makes it an eco-friendly fishing technique
Source: PLoS One. 2020 Aug 21;15(8):e0237819. doi: 10.1371/journal.pone.0237819 (PMC7442244; doi:10.1371/journal.pone.0237819)

Supplementary material 1: detailed technical settings of the two different traps

Lightweight trap – 13 kg

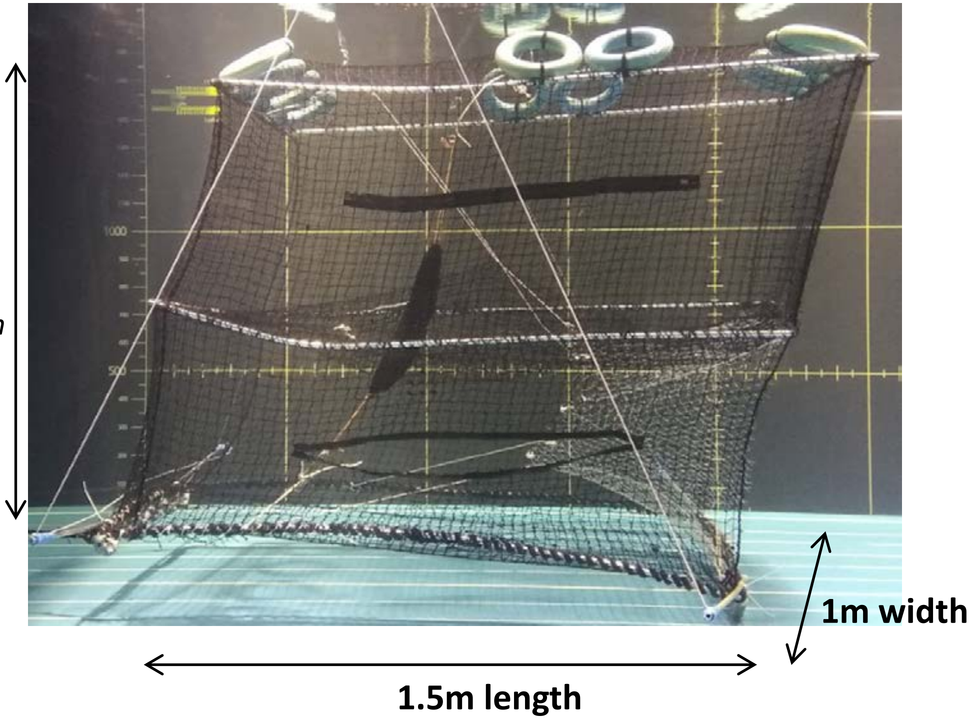

Heavy trap – 31 kg

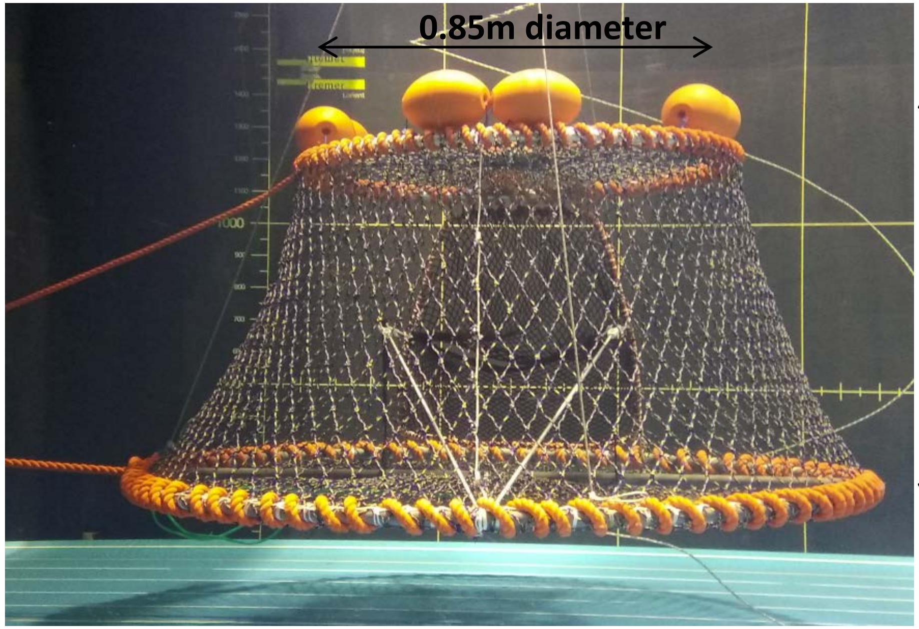

Supplement: S1 Fig — (PDF) [file pone.0237819.s001.pdf]
