# Supplementary material for: The low impact of fish traps on the seabed makes it an eco-friendly fishing technique
Source: PLoS One. 2020 Aug 21;15(8):e0237819. doi: 10.1371/journal.pone.0237819 (PMC7442244; doi:10.1371/journal.pone.0237819)

Supplementary material 2

Current directions in degree measured prior the trap impact experiment to determine the settlement direction of the trap line.


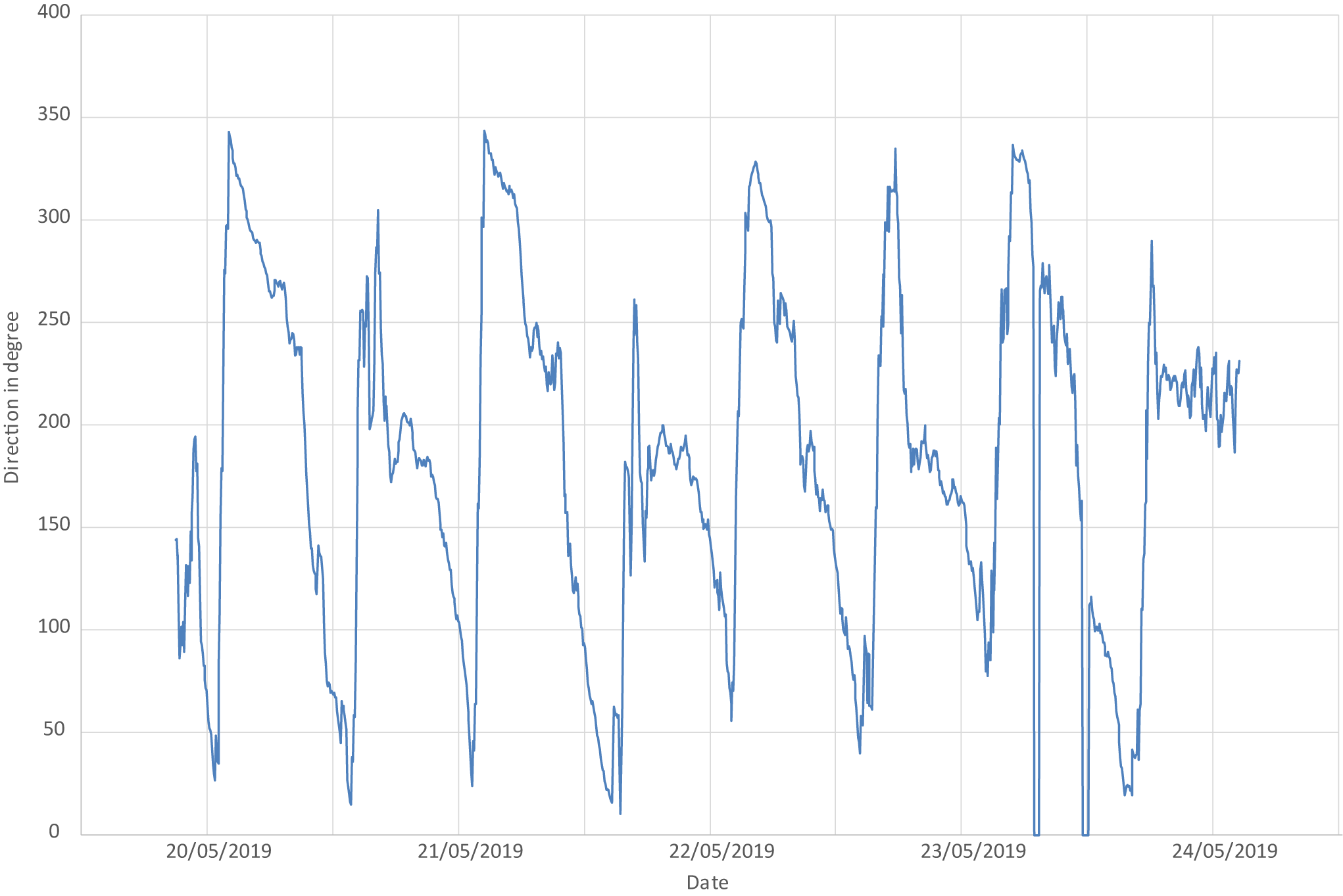

Supplement: S2 Fig — (DOCX) [file pone.0237819.s002.docx]
