# Supplementary material for: The low impact of fish traps on the seabed makes it an eco-friendly fishing technique
Source: PLoS One. 2020 Aug 21;15(8):e0237819. doi: 10.1371/journal.pone.0237819 (PMC7442244; doi:10.1371/journal.pone.0237819)

Supplementary material 3

1. the chessboard used to correct lens distortion; b) the cube used for 3D calibration.


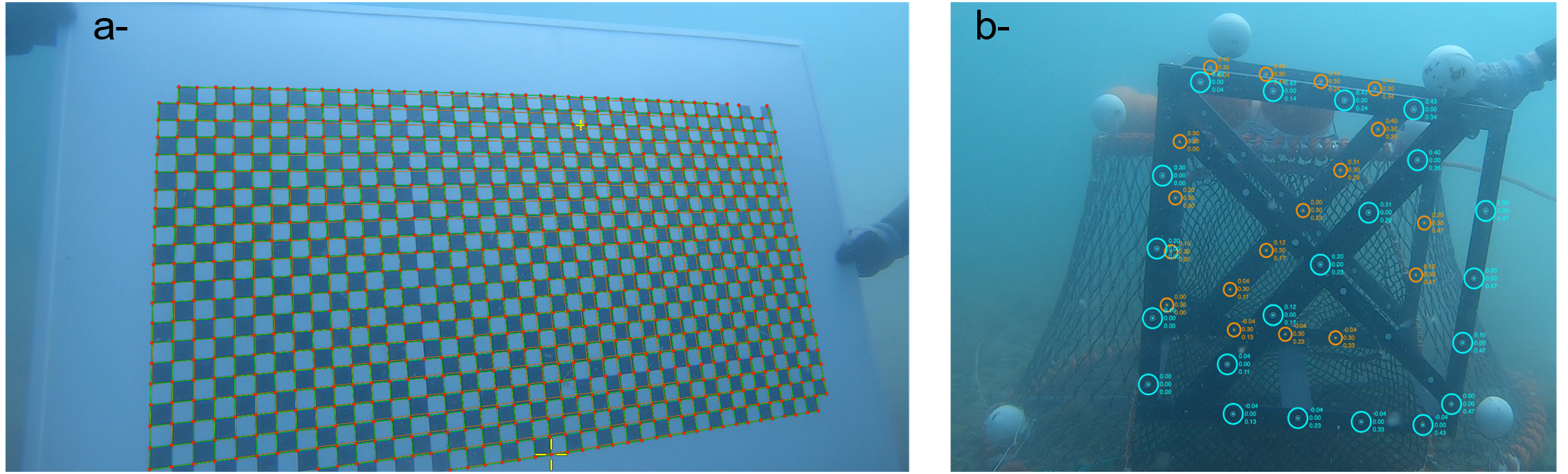

Supplement: S3 Fig — (DOCX) [file pone.0237819.s003.docx]
